# Supplementary material for: MetaRibo-Seq measures translation in microbiomes
Source: Nat Commun. 2020 Jun 29;11:3268. doi: 10.1038/s41467-020-17081-z (PMC7324362; doi:10.1038/s41467-020-17081-z)
Supplement: Supplementary file 10 — Supplementary Data 7 [file 41467_2020_17081_MOESM10_ESM.zip › File2/Confidence_High_Taxonomy/192160_out.krona.html]

Javascript must be enabled to view this page.

members
magnitude
magnitudeUnassigned
count
unassigned
taxon
rank

192160\_out

2

2
2
superkingdom

2
976
phylum

class
200643
2

171549
order
2

815
family
2

2
genus
816

1
1896974

SRS1041092\_contig\_number\_14047
species

1
246787
species

SRS893230\_contig\_number\_5961
